# Report of the International Society for Zinc Biology 5th Meeting, in Collaboration with Zinc-Net (COST Action TD1304)—UCLan Campus, Pyla, Cyprus

**DOI:** 10.3390/ijms18122518

**Published:** 2017-11-24

**Authors:** Nicola M. Lowe, Victoria Hall Moran

**Affiliations:** 1 International Institute of Nutritional Sciences, and Applied Food Safety Studies, Faculty of Health and Wellbeing, University of Central Lancashire, Preston PR1 2HE, UK; 2 School of Community Health & Midwifery, Faculty of Health and Wellbeing, University of Central Lancashire, Preston PR1 2HE, UK; vlmoran@uclan.ac.uk

**Keywords:** zinc, conference report, Zinc-Net, ISZB, COST Action

## Abstract

From 18 to 22 June 2017, the fifth biennial meeting of the International Society for Zinc Biology was held in conjunction with the final dissemination meeting of the Network for the Biology of Zinc (Zinc-Net) at the University of Central Lancashire, Cyprus campus. The meeting attracted over 160 participants, had 17 scientific symposia, 4 plenary speakers and 2 poster discussion sessions. In this report, we give an overview of the key themes of the meeting and some of the highlights from the scientific programme.

## 1. Introduction

The meeting was jointly hosted as a partnership between the International Society for Zinc Biology (ISZB) and the Network for the Biology of Zinc (Zinc-Net), a European Union Framework Programme, Horizon 2020-funded Collaboration in Science and Technology (COST) Action. It was the fifth biennial meeting of the ISZB, which is now celebrating a decade of achievements. The society was established to allow unique interactions between scientists interested in zinc biology and has been successful in fostering links between the chemical, biological and clinical fields of zinc biology [1]. Zinc-Net was launched in 2013 and has been funded for 4 years. It is comprised of over 200 scientists from 26 different European countries, as well as Australia. The overall mission of the network was to create a multi-disciplinary research platform that brings together expertise from research groups throughout the COST countries and beyond, as well as to stimulate and accelerate new, innovative and high-impact scientific research [2]. The clear synergy between these two organisations created a stimulating forum for lively scientific debate over the 5 days of the conference.

## 2. Meeting Overview

### 2.1. Zinc-Net Celebration Symposium

The meeting was the final major event of the 4 year COST Action, Zinc-Net. Therefore, it began with a symposium that celebrated the achievements of Zinc-Net, with presentations from each of the theme leaders in Chemical Biology, Biomarker Discovery and Clinical Coordination. A key note lecture by Professor Janet King (Chair of the International Zinc Nutrition Consultative Group, Children’s Hospital Oakland Research Institute, Oakland, CA, USA), and Professor Nicola Lowe (Chair of the Zinc-Net Management Committee, University of Central Lancashire, Preston, UK), with comments by Professor Mukhtiar Zaman (Lady Reading Hospital, Khyber Pakhtunkhwa, Pakistan), placed zinc research into a global context, highlighting the extent of zinc deficiency worldwide, with the highest prevalence of over 40% of the population in low- and middle-income countries. A display of posters showcasing some of the short-term scientific missions (laboratory exchange visits) that had been undertaken by early career researchers in the network were available to view [3].

### 2.2. Keynote Lectures

The first keynote lecture of the main conference was given by the Founding President of the ISZB, Professor Glen K. Andrews (University of Kansas School of Medicine, Kansas City, MO, USA). He was introduced by the current president, Dr. Kathryn Taylor (Cardiff University, Wales, UK). Professor Andrews’s lecture recognised the contributions of key scientists whose research was of fundamental importance to the advancement of the field of zinc. These included the discovery of the metallothionein (MT) and the recognition that these proteins have a wide species distribution and are inducible by metals, the elucidation of a zinc-sensing mechanism of transcriptional regulation of MT genes, and the discovery of the Slc30a (ZnT) and Slc39a (ZIP) families of zinc transporters. These findings informed further studies of the mechanisms of zinc-dependent regulation of the ZIP proteins and their important physiological role. Each of these discoveries led to thousands of subsequent research publications and provided the foundation for much of the current research into the biology of zinc [4].

The second keynote lecture, by Professor Hidenori Ichijo (Graduate School of Pharmaceutical Sciences, U-Tokyo, Japan) discussed the physiological and pathophysiological roles of copper/zinc superoxide dismutase (SOD1) under conditions of zinc deficiency. The importance of understanding the molecular mechanism of zinc homeostasis in living organisms is highlighted by zinc’s essentiality in a wide variety of biological processes and the consequences of zinc deficiency in human health and disease. Ichijo’s group recently reported that SOD1 is one of the key factors to regulate cellular zinc homeostasis under zinc-deficient conditions. In zinc deficiency, SOD1 adopts an abnormal conformation and evokes endoplasmic reticulum (ER) stress through specific interaction with Derlin-1, a component of ER-associated degradation (ERAD) machinery, leading to the restoration of cellular homeostasis. Intriguingly, they found that wild-type SOD1 under zinc-deficient conditions and over 100 types of amyotrophic lateral sclerosis (ALS)-linked SOD1 mutants share the common aberrant conformation, suggesting that wild-type SOD1 has a potential to exert neuronal toxicity under stress conditions. Professor Ichijo’s group have performed genome-wide siRNA screening to identify mediators of the conformational alteration in wild-type SOD1 under conditions of zinc deficiency, and he discussed the physiological and pathophysiological implications [5].

Professor Stephen J. Lippard’s (Massachusetts Institute of Technology, Cambridge, MA, USA) keynote discussed the role of zinc probes as tools in the study of mobile Zn^2+^; these serve as signaling agents in a number of biological processes, including neurotransmission. A new class of hybrid fluorescent sensors that facilitate the tunability of small molecule probes and the targetability of protein-based sensors was described, which can detect exogenous Zn^2+^ and endogenous mobile Zn^2+^ in response to reactive nitrogen species in live cells. The functional role of Zn^2+^ in the olfactory system was discussed, including results obtained from fluorescence imaging and electrophysiology recordings of live animals exposed to a variety of odours. Synaptically released mobile Zn^2+^ attenuates excitatory postsynaptic currents carried by *N*-methyl-d-aspartate (NMDA) receptors in the olfactory bulb, thus attenuating sensory input gain [6].

Despite increasing access to sufficient food for all and significant achievements in reducing global hunger, micronutrient deficiencies (“hidden hunger”), especially zinc deficiency, still remain a major public health problem in the world. An inadequate daily intake of zinc is the major reason for the problem, particularly in the developing world, where extensive amounts of cereals are consumed with very low concentrations of bioavailable zinc. In the final keynote lecture, Professor Ismail Cakmak (Sabanci University, Istanbul, Turkey) described several agricultural strategies that are known to improve grain-zinc concentration, including conventional plant breeding, genetic engineering, and plant nutrition-based agronomy. In recent years, there has been an increase in the number of published reports showing that the maintenance of the high pool of zinc in the leaf tissue during the reproductive growth stage is required to achieve desirable concentrations of zinc in grains for human nutrition. Field experiments conducted in different countries under the HarvestZinc project [4] on maize, wheat and rice demonstrated that a foliar spray of zinc and other micronutrients such as iodine and selenium results in substantial increases in concentrations of those micronutrients, in both the whole grain and the endosperm. In addition, foods made from cereal grains that have been biofortified agronomically with micronutrients, such as bread and cookies, also had elevated micronutrient concentrations, evidencing the stability of the micronutrients in products. Consuming agronomically biofortified foods is expected to result in a significant contribution to human nutrition with the potential to impact on micronutrient deficiencies worldwide [7].

## 3. Research Themes of the Meeting

The 17 scientific symposia that comprised the meeting can be grouped into three main themes: zinc in health and disease, zinc signalling, and zinc proteins and transporters. A summary of each is reported below and further up-to-date information on key topics in zinc research has been published by Rink [8] and zinc signalling by Fukada and Kambe [9].

### 3.1. Zinc in Health and Disease

The role of zinc in immunity and infectious disease was explored and debated. Zinc is recognized as an important metal ion in relation to nutritional immunity, a process by which the immune system withholds micronutrients from potential invaders. An understanding of the underlying mechanisms by which host immune defences manipulate metal levels to attack invading microbes by metal-restriction and/or exposure to metal-excess may have considerable clinical significance. Giving *Campylobacter jejuni* (Cj), a common cause of acute human gastroenteritis, as an example of an important foodborne pathogen that targets different host niches with different metal challenges, how Cj adapts to different metal stresses within its different hosts was described. Exploring the functions and mechanisms of the zinc handling systems in *Campylobacter* will expand our understanding of how they contribute to infections. Zinc deficiency is linked to an increased susceptibility to bacterial infection, such as *Streptococcus pyogenes* (Group A *Streptococcus*—GAS), a Gram-positive human pathogen responsible for a wide spectrum of diseases ranging from pharyngitis and impetigo, to severe invasive diseases including necrotizing fasciitis and streptococcal toxic shock-like syndrome. It was demonstrated that zinc homeostasis is an important contributor to GAS pathogenesis and innate immune defence against infection. Strategies to manipulate zinc homeostasis in order to reduce GAS infection were discussed.

Zinc-based therapeutics were explored in relation to cognitive disorders, cancer and cardiovascular disease. One of the critical cell processes that becomes dysregulated with age and also in disease, and which participates both directly and indirectly in cognitive function, is metal homeostasis and the neurochemistry of metalloproteins. This is particularly true for zinc, for which 10–15% of brain zinc exists in a chelatable form, primarily within synaptic vesicles at glutamatergic synapses, highlighting its potential importance in synaptic plasticity/cognition. Zinc dyshomeostasis has been implicated in dementia and autism spectrum disorders. Taken together with other supporting data in the literature, this demonstrates a critical role for zinc in cognitive function, and that it may be a therapeutic target for improving functional outcomes in health and disease.

A significant body of evidence has shown that zinc plays important roles in metabolism and the development of metabolic disease. Zinc has a role in insulin secretion, insulin signalling and subsequent glucose metabolism. Low zinc status also promotes inflammatory stress, and using mouse models of atherosclerosis, vascular inflammation and plaque formation have been shown to be enhanced by marginal zinc deficiency. Increased intestinal permeability plays an important role in the onset of a variety of chronic inflammatory conditions and metabolic diseases, and zinc has been found to improve gut barrier integrity in vitro. In humans, a low-zinc diet is associated with a decrease in fatty acid desaturase enzyme 1 (FADS1) activity, lowered arachidonic acid incorporation into lipid subclasses, and an increase in DNA strand breaks, suggesting that FADS1 activity and DNA strand breaks respond to small changes in dietary zinc that may be provided by food fortification programmes.

### 3.2. Zinc Signalling

The roles of zinc in modulating cellular function in various disease states emerged as a key theme of this meeting. These included new advances in understanding how disrupted Zn^2+^ homeostasis in chronic heart failure is linked to dysregulated intracellular Ca^2+^ responses, resulting in leakage of Ca^2+^ from the sarcoplasmic reticulum in cardiac tissue. Research describing the pathophysiological role of zinc in neurological disorders provided insights into possible novel therapeutic approaches. Examples included the role of zinc in triggering neuronal apoptosis and blocking optic nerve regeneration after injury, as well as the role of extracellular zinc in the modulation of the cytokine-induced pro-inflammatory response following brain ischaemia, which may contribute to impaired memory function. New research examining the pathophysiological role of extracellular Zn^2+^ in cognitive decline with aging was received with interest. Highlights within this theme also included new understandings of the molecular mechanisms for maintaining cellular zinc homeostasis and the use of novel zinc sensors, which can be activated by UV light or enzymes for the study of the dynamics of cellular “free” zinc.

### 3.3. Zinc Proteins and Transporters

One of the most exciting areas of research over the last decade has been the discovery of the zinc transporter families ZIP and ZnT and the elucidation of their role in the control of cellular zinc homeostasis. Within this theme, the relationship between the loss of ZnT2 function within paneth cells and intestinal dysbiosis was discussed. This research revealed that genetic polymorphisms that influence the ZnT2 transporter function might lead to clinically relevant shifts in the intestinal microbiome of preterm infants, which is a fascinating new area of research relating to infant nutrition. Similarly, ZIP7 plays a critical role in ER function within connective tissue cells, such that a loss of the function of this transporter results in inhibited cell proliferation, preventing proper dermis formation. Highlights within this theme included potential new therapies for the treatment of cancer, linked to the inhibition of mitosis through the selective blocking of ZIP transporters. Additionally, the unexpected association between genetic mutations in ZIP13, zinc homeostasis and beige adipocyte biogenesis may contribute to new therapies for obesity and metabolic syndrome.

Other zinc binding proteins also shared the limelight in this theme. Notably, the influence of zinc binding on protein folding and aggregation may have deleterious consequences if intracellular zinc homeostasis becomes imbalanced. Also discussed was the mechanism of the activation of the zinc-requiring ectoenzymes, defined as secretory, membrane bound, and organelle-resident enzymes, which play pivotal roles in numerous biological responses. One such example is tissue non-specific alkaline phosphatase, which is activated in a two-step process involving ZnT transporters.

## 4. Focus on Early Career Researchers

The conference was attended by over 160 scientists, including early career researchers (ECRs), many of whom presented posters in one of the two evening poster sessions. Both Zinc-Net and ISZB place a strong emphasis on providing training opportunities, capacity building and support for the next generation of zinc biologists. In a competitive process, ECRs were invited to present their research in two special symposia showcasing the work of these up and coming young scientists in this exciting field.

## 5. Final Remarks

Much of the research presented at this meeting had never been presented or published before. The meeting had a strict embargo on the photographing of slides without the presenters’ permission, and the abstracts, although made available to all participants, were not to be published in proceedings of the meeting. However, it was gratifying to observe that the atmosphere within the meeting was extremely open and collegiate, with new collaborations initiated and many animated discussions during the social, networking and poster events. The zinc research community is clearly thriving, and it is exciting to be a part of it.

## Abbreviations

FADS1	Fatty acid desaturase enzyme 1
NMDA	*N*-Methyl-d-aspartate
ERAD	ER-associated degradation
ISZB	International Society of Zinc Biology
COST	Collaboration in Science and Technology
SOD	Superoxide dismutase
ECR	Early career researcher
MT	Metallothionein
ER	Endoplasmic reticulum
Cj	*Campylobacter jejuni*
